# Assessing the Feasibility, Usability, Acceptability, and Efficacy of an AI Chatbot for Sleep Promotion: Quasi-Experimental Study

**DOI:** 10.2196/84023

**Published:** 2026-02-03

**Authors:** Xiaoyue Liu, Jingchen Liu

**Affiliations:** 1 New York University New York, NY United States; 2 Columbia University New York, NY United States

**Keywords:** artificial intelligence, AI, large language models, chatbot, feasibility, usability, acceptability, sleep

## Abstract

**Background:**

Poor sleep is a concerning public health problem in the United States. Previous sleep interventions often face barriers such as high costs, limited accessibility, and low user engagement. Recent advancements in artificial intelligence (AI) technologies offer a novel approach to overcoming these limitations. In response, our team developed a prototype AI sleep chatbot powered by a large language model to deliver personalized, accessible sleep support.

**Objective:**

This study aimed to examine the feasibility, usability, acceptability, and preliminary efficacy of the AI chatbot for sleep promotion.

**Methods:**

We conducted a quasi-experimental, single-group study with adults in the United States aged 18 to 75 years who self-reported poor sleep. The chatbot was integrated into a commercially available messaging app. Participants were asked to engage with a virtual sleep therapist via texting over 2 weeks. The chatbot provided ongoing, individualized sleep guidance and adapted recommendations based on participants’ prior conversations. Feasibility, usability, and acceptability were descriptively summarized. Sleep was assessed using questionnaires before and after the intervention.

**Results:**

Of the 107 adults who enrolled in the study, 88 (82.2%) completed chatbot registration. Among these 88 participants, 65 (73.9%) initiated interactions, and 44 (50%) completed the 2-week intervention. The final analysis included 42 adults (mean age 36, SD 11 years; n=12, 28.6% male). On average, participants engaged with the chatbot for 58 (SD 42) minutes, with each chat session lasting approximately 9 (SD 6) minutes. Most reported favorable experiences with the chatbot. The average usability score was 85.2 (SD 10.7) out of 100, which was well above the benchmark of 68. The chatbot was rated as highly acceptable, with a satisfaction score of 27.3 (SD 4.1) out of 32. All participants perceived the chatbot as effective, with ratings ranging from “slightly effective” to “extremely effective.” The preliminary evidence showed improved sleep outcomes after chatbot use: total sleep time increased by 1.4 hours (*P*<.001); sleep onset latency decreased by 30.9 minutes (*P*<.001); sleep efficiency increased by 7.8% (*P*=.007); and scores improved for perceived sleep quality (mean difference [MD] −5.4; *P*<.001), insomnia severity (MD −7.9; *P*<.001), daytime sleepiness (MD −4.7; *P*<.001), and sleep hygiene skills (MD −13.2; *P*<.001). No significant change was observed in sleep environment (MD −1.1; *P*=.16).

**Conclusions:**

Our AI chatbot demonstrated satisfactory feasibility, usability, and acceptability. Improvements were observed following chatbot use, although causality cannot be established. These findings highlight the potential of integrating state-of-the-art large language models into behavioral interventions for sleep promotion. Future research should include objective sleep measurements and conduct randomized controlled trials to validate the study findings. If confirmed, this AI chatbot could be scaled to support sleep health on a broader level.

## Introduction

Poor sleep is a major public health concern in the United States. Major guidelines recommend that adults sleep at least 7 hours per night [1,2]. Over 25% of adults in the United States do not meet this recommendation, and 14.5% had difficulty falling asleep on most days or every day over the previous month [3]. Deviations from optimal sleep duration and quality play a crucial role in the development and progression of various health conditions. For example, adults with chronic experience of poor sleep have a higher risk of developing depression later in life [4]. Similarly, extensive epidemiological evidence links insufficient sleep to an increased risk of cardiovascular disease [5-7]. Adults with poor sleep quality have a 1.38-fold higher mortality rate in comparison to those with good sleep quality [8]. The ramifications extend beyond individual health. Poor sleep directly links to reduced productivity, lower work performance, and increased health care use [9]. On average, poor sleep is associated with US $3400 to US $5200 in additional health care expenditures per person [10]. At the population level, insufficient sleep causes the United States to lose approximately 1.23 million workdays annually, with projected economic costs of up to US $456 billion by 2030 [11]. Despite these serious consequences, health care providers rarely target sleep as an approach to improve health outcomes [12]. Therefore, implementing an effective intervention to alleviate the public health burden resulting from poor sleep is a must.

Existing behavioral interventions such as cognitive behavioral therapy for insomnia (CBT-I) have proven effective in improving sleep outcomes. However, their widespread use is primarily constrained by high costs, limited accessibility, and a lack of personalization. These barriers hinder the broad adoption of sleep interventions. Consequently, digital tools have emerged as a solution for enhancing sleep health. There has been growing interest in incorporating artificial intelligence (AI) into treatments. AI-based technologies, particularly conversational tools, have shown growing evidence of health behavior change. A recent systematic review found that chatbots can promote healthy lifestyles, support smoking cessation, improve medication adherence, and reduce substance use [13]. By simulating humanlike conversations, chatbots deliver on-demand, tailored support that is typically not achievable with traditional in-person or telehealth care. Although limited, emerging research has suggested the potential benefit of using chatbots in improving sleep [14]. Nurses who used a chatbot that offered care support and coping assistance reported better sleep quality after 6 weeks in comparison to the control group [15]. Another study used a chatbot to gather conversations about sleep from parents of preterm and term infants to better understand their personal experiences [16]. Yet, most chatbot interventions still rely on decision tree algorithms, which often struggle to interpret conversational nuances and context. Recent breakthroughs in generative AI offer novel alternatives to conventional behavioral interventions. Generative AI systems learn patterns from large datasets to produce new, realistic, and coherent content that resembles human communication. A prominent example is large language models (LLMs), an advanced form of generative AI that uses deep learning to comprehend and generate natural-sounding responses. Currently, there is scarce or no evidence on integrating state-of-the-art LLMs in sleep interventions. In response, we developed a prototype AI chatbot using LLMs that provides responses specifically to promote sleep. This study sought to (1) evaluate the feasibility, usability, and acceptability of the AI chatbot; and (2) test the preliminary efficacy of the AI chatbot on improving sleep outcomes.

## Methods

### Study Design and Sample

We used a quasi-experimental 1-group design. Using a convenience sampling approach, participants who self-reported poor sleep in the previous month were recruited nationwide in the United States. Our inclusion criteria were (1) age of 18 to 75 years, (2) a raw score of 23 or higher on the Patient-Reported Outcomes Measurement Information System Sleep Disturbance [17], (3) ownership of a smartphone and ability to access the internet, (4) willingness and ability to participate through texting on a smartphone, and (5) ability to read and understand English. Individuals were excluded if they were pregnant, lactating, or concurrently participating in other research studies focused on lifestyle modifications.

### Generative AI Technology

**Figure 1:**
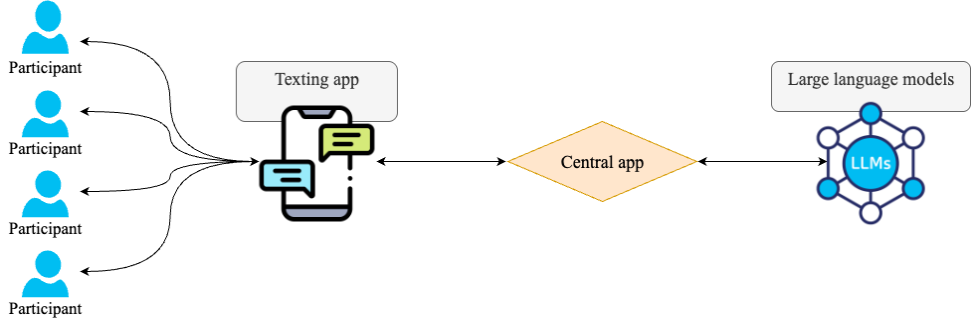
Workflow of the generative artificial intelligence sleep chatbot. LLM: large language model.

Figure 1 illustrates the automated workflow of the generative AI technology. The main body of the infrastructure comprises a central app designed by our study team, which communicates with a messaging app and LLMs via an application programming interface.

We created an AI chatbot on the messaging app platform that allowed participants to exchange texts as if they were interacting with a real sleep therapist. The chat operates on a commercially available messaging platform, which has a wide user base in the United States and is available to both iOS and Android smartphones. When a participant sends a message to the chatbot, the message is pushed to the central app. The central app is entirely controlled by our study team. It processes incoming messages based on the participant’s chat history and then sends a query to the LLMs. We used Gemini-1.5-pro-001 (Google) and the CBT-I manual as the context for each prompt, with guidance focused on the following treatment components: stimulus control (strengthen the bed as a cue for sleep), sleep hygiene practices (promote healthy sleep habits), relaxation training (improve techniques that relax the mind and body), and cognitive restructuring (change negative thoughts and beliefs about sleep). For safety concerns, the prompts were designed to exclude sleep restriction therapy, a key CBT-I element that involves reducing time in bed. The responses from the LLMs then return to the central app, which subsequently forwards them to the participant through the chatbot on the messaging app.

To ensure participant safety, we implemented several safeguards and configured the LLMs with a reasonably high safety setting. Details of the model configuration and safety parameters are provided in Multimedia Appendix 1. All conversational inputs and outputs were screened for hate speech, harassment, sexual content, self-harm, or medical misinformation. Before deployment, we prompted the LLMs to avoid providing medical diagnoses or medication advice and stress tested them with adversarial prompts. Participants were instructed not to share personal identifiers when interacting with the chatbot. Any inadvertent disclosures were immediately removed from the chat history by the study team. Communications with the LLMs and with the third-party messaging app were encrypted in transit using HTTPS. Our team monitored the conversational dialogues daily throughout the intervention phase to ensure that the advice delivered to participants was appropriate and safe, and to promptly identify any conversations necessitating immediate action (eg, indication of self-harm).

### Intervention Description

**Figure 2:**
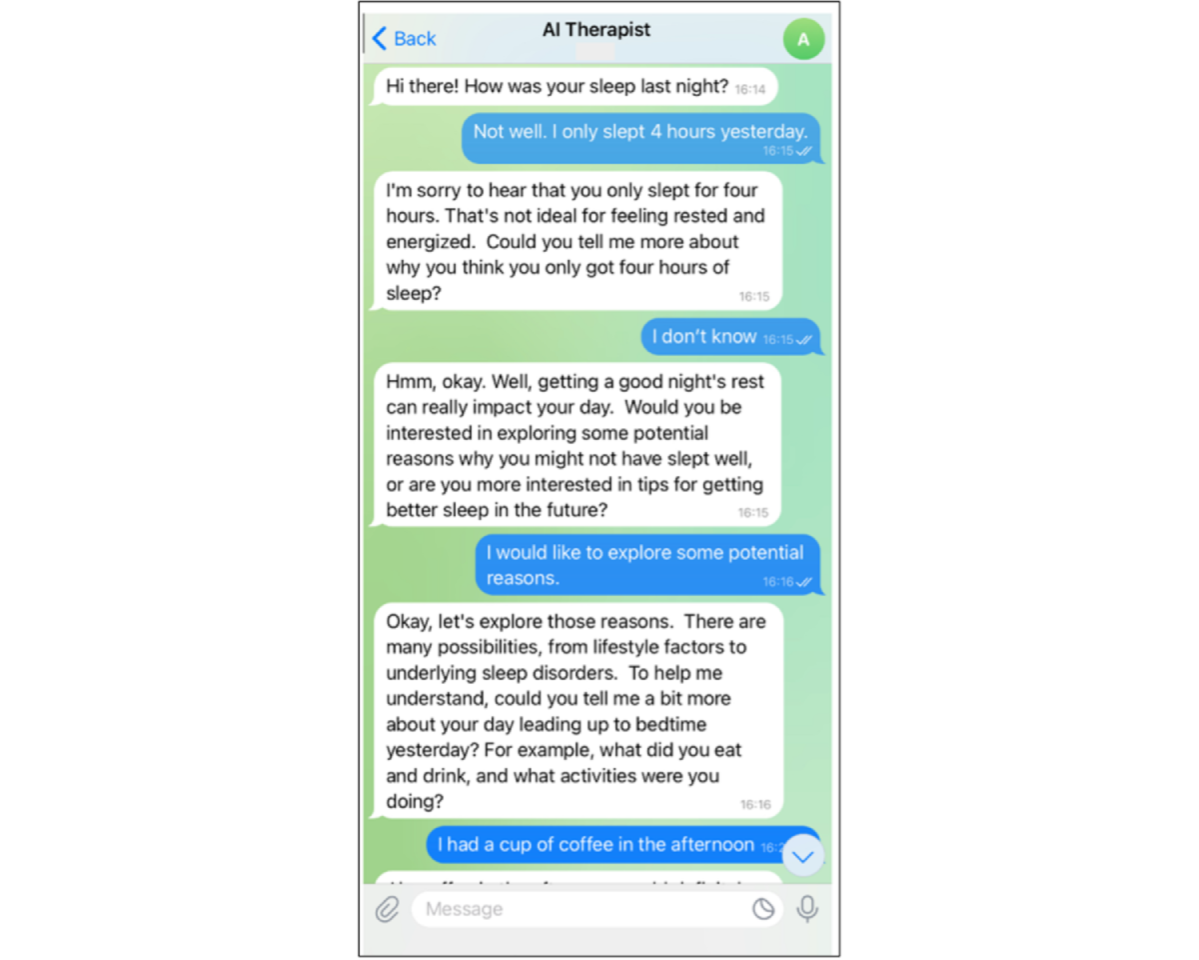
Example of conversations between the chatbot and participant.

The intervention lasted 2 weeks and was delivered through the chatbot designed to function as a virtual sleep therapist. It created individualized treatment plans tailored to each participant’s needs and feedback. By applying motivational interviewing techniques, the chatbot explored underlying causes of sleep disturbances and identified appropriate solutions aligned with key components of CBT-I. If certain issues were unresolved, the chatbot conducted further analysis using LLMs to examine additional strategies. The chatbot also retained memory of previous conversations, allowing it to follow up with participants, reinforce recommendations, and detect any challenges in adherence. An example of conversations between the AI chatbot and a participant is illustrated in Figure 2.

### Ethical Considerations

This study was approved by the New York University Institutional Review Board (FY2024-9037). Informed consent was obtained from all participants before any procedures. Participation was voluntary. Individuals could withdraw at any time or skip any question without penalty. Each participant was assigned a unique study ID. Data were securely stored on university-managed, access-controlled devices and systems with access limited to study personnel. The university devices and systems encrypt the data at rest. We did not have a data processing agreement with the third-party messaging app. However, participants were informed during the consent procedures that the study required the use of a third-party messaging platform. All personal identifiers were removed before analysis. Deidentified analytic files will be retained for 5 years after publication and then permanently deleted.

### Procedures

After providing informed consent, participants registered for the chatbot on a third-party messaging app. They then completed a prestudy survey that inquired about their sociodemographic information, clinical background, and sleep characteristics. The intervention began when participants initiated conversations with the chatbot via texting. To protect confidentiality and privacy, the chatbot only responded to those who initiated contact. Participants were instructed to text with the chatbot at least every 3 days for 2 weeks, with each chat session lasting approximately 3 to 5 minutes. We classified individuals as dropouts if they interacted with the chatbot fewer than 4 days in total or had an average daily duration of engagement of less than 1 minute during the intervention period. In this study, participants could chat as frequently or as long as they preferred. After the initial conversations, the chatbot followed up with participants if no interactions were detected within 72 hours. In subsequent sessions, the chatbot checked in with participants about their sleep concerns, reinforced recommended sleep promotion strategies, and suggested new ones when necessary. Finally, participants completed a poststudy survey upon concluding the intervention.

### Measurements

#### Participant Flow

Feasibility was assessed based on the following metrics: (1) the proportion of individuals who registered for the chatbot; (2) the proportion of registered participants who initiated engagement with the chatbot; (3) the proportion of active participants who completed the 2-week intervention; and (4) chatbot engagement, including the total number of days interacting with the chatbot, total duration of chat sessions, and average duration per session.

#### Usability

Usability was measured via the Chatbot Usability Questionnaire (CUQ) [18]. This instrument consists of 16 items scored on a 5-point Likert-type scale ranging from 1 (“strongly disagree”) to 5 (“strongly agree”). It measures personality, onboarding, user experience, and error handling of a chatbot. Odd-numbered items assess positive aspects, whereas even-numbered items assess negative aspects. Responses to all items are summed and then converted to a 100-point scale to be comparable with the System Usability Scale [19]. A total score of 68 is typically used as the threshold for acceptable usability [20].

#### Acceptability

Acceptability was measured using the adapted Client Satisfaction Questionnaire (CSQ) [21] and 6 questions developed by the study team. The original CSQ is an instrument to assess a client’s overall level of satisfaction [21]. We revised the wording to make the questions fit with the study context. Each item is rated on a 4-point Likert scale from 1 (“poor”) to 4 (“excellent”). The total score ranges from 8 to 32, with a higher number indicating greater satisfaction. We also developed the following questions to evaluate the participants’ experiences: (1) “What changes have you noticed about your sleep after engaging with the chatbot?”; (2) “How effective were the chatbot’s tips for improving your sleep?” (rated from 1=“Not effective” to 5=“Extremely effective”); (3) “Did the chatbot offer personalized advice based on your input?” (rated from 1=“Never” to 5=“Always personalized”); (4) “How well did the chatbot adapt to your sleep concerns?” (rated from 1=“Not at all” to 5=“Very well”); (5) “How well did the chatbot remember your preferences in follow-up conversations?” (rated from 1=“Not at all” to 5=“Very well”); and (6) “Did you feel supported by the chatbot during your interactions?” (rated from 1=“Not at all” to 5=“Very well”). These self-developed items were validated scales, and they were intended only for formative evaluation.

#### Preliminary Efficacy

The preliminary efficacy of the chatbot was examined using several metrics.

Habitual sleep patterns and perceived sleep quality were measured using the Pittsburgh Sleep Quality Index [22]. This 19-item instrument evaluates sleep quality and disturbances over the previous month. The items cover 7 components: subjective sleep quality, sleep onset, sleep duration, habitual sleep efficiency, sleep disturbances, use of sleep medication, and daytime dysfunction. Each component score ranges from 0 to 3. These components are then summed to generate a global score ranging from 0 to 21, where a cutoff score of 5 or higher indicates poor sleep quality. Additionally, we used items from the Pittsburgh Sleep Quality Index to assess 3 habitual sleep patterns, which included sleep onset, sleep duration, and sleep efficiency. Sleep onset was measured using the question “How long has it usually taken you to fall asleep each night?” Sleep duration was measured using the question “How many hours of actual sleep did you get at night?” Sleep efficiency was calculated by dividing sleep duration by time spent in bed and multiplying the result by 100.

Insomnia severity was measured using the Insomnia Severity Index [23]. The Insomnia Severity Index assesses the severity and impact of insomnia over the previous 2 weeks. It comprises 7 items, with the score of each item evaluated on a 5-point Likert-type scale. The total score ranges from 0 to 28, and a higher score indicates more severe insomnia symptoms. A total score over 14 indicates clinical insomnia.

Daytime sleepiness was measured using the Epworth Sleepiness Scale. The Epworth Sleepiness Scale is an 8-item, 4-point Likert scale that measures the likelihood of dozing off or falling asleep in 8 common life situations [24]. Participants who had not experienced some of the situations were asked to estimate how each might affect them. A total score over 10 indicates excessive daytime sleepiness.

Sleep hygiene was measured using the Sleep Hygiene Index [25]. The Sleep Hygiene Index is a 13-item, 5-point Likert scale to evaluate the frequency of participants engaging in specific sleep behaviors. The sum of each item score yields a global score ranging from 0 to 52, with a higher score representing more maladaptive behaviors that compromise sleep hygiene.

Sleep environment was measured using the Assessment of Sleep Environment [26]. This instrument includes 13 items that quantify the impact of light and dark, noise, smell, humidity, comfort of the sleeping surface and pillows, and safety on an individual’s sleep. Each item is rated on a scale from 0 (“strongly disagree”) to 3 (“strongly agree”) and can be summed to obtain a total score. A higher total score suggests living in a poorer sleep environment.

### Statistical Analysis

Sociodemographic and clinical characteristics were summarized as mean and SD for continuous variables or as count and percentage for categorical variables. Conversational dialogue data were analyzed descriptively to assess the level of engagement. Usability and acceptability measures were used to visualize bar plots. Changes in each sleep measure before and after the intervention were assessed for normality using the Shapiro-Wilk test and visualized using histograms and *Q*-*Q* plots. Outliers were identified and removed. Depending on whether normality assumptions were met, pretest-posttest differences in each sleep variable were evaluated using either 2-tailed paired *t* tests or Wilcoxon signed rank tests. We also computed the Cohen *d* to estimate the effect size associated with each pretest-posttest change. Additionally, we used scatterplots to visualize individual changes in sleep variables, with posttest values on the x-axis and pretest values on the y-axis. We also calculated the proportion of participants who showed improvements in each sleep measure after the intervention. All statistical analyses were performed using R (version 4.5.0; R Foundation for Statistical Computing) [27]. The significance threshold was set at α=.05.

## Results

### Feasibility

**Figure 3:**
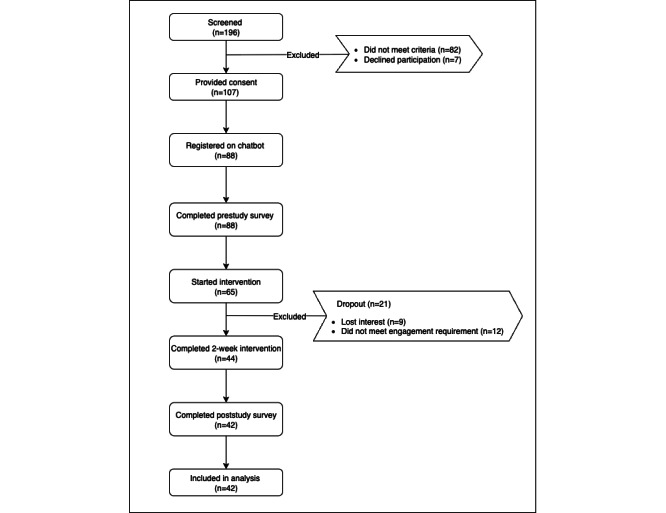
Participant flow diagram.

Figure 3 presents the participant flow diagram for this study. A total of 196 individuals were screened for eligibility. Of those 196 individuals, 107 (54.6%) adults met the study criteria and provided informed consent. Of these 107 adults, 88 (82.2%) completed chatbot registration and the prestudy survey. A total of 60.7% (65/107) of the participants initiated conversations with the chatbot. In total, 41.1% (44/107) of the participants completed the 2-week intervention. Of these 44 participants, 42 (95.5%) completed the poststudy survey and were included in the final analysis. The completion rates for all outcome variables were 100% without missing data. In Multimedia Appendix 2, we compare baseline characteristics between participants who completed the intervention and those who did not start it or did not complete it. No statistically significant differences were found between the 2 groups.

### Sample Characteristics

Table 1 summarizes the key sociodemographic and clinical characteristics of the 42 study participants. Their mean age was 36 (SD 11) years, with a range from 22 to 59 years. A total of 28.6% (12/42) of the participants were male, and 61.9% (26/42) were White individuals. In total, 28.6% (12/42) reported taking sleep medications at night. A total of 81.0% (34/42) of the participants slept less than 7 hours per night. Nearly all (41/42, 97.6%) reported having poor sleep quality. In total, 73.8% (31/42) met the criteria for clinical insomnia, and 47.6% (20/42) experienced excessive daytime sleepiness.

**Table 1:** Characteristics of the study sample (n=42).

Characteristic				Values
**Sociodemographic and clinical characteristics**				
	Age (y), mean (SD)			36 (11)
	**Sex, n (%)**			
		Male	12 (28.6)	
		Female	30 (71.4)	
	**Race, n (%)**			
		Non-White	16 (38.1)	
		White	26 (61.9)	
	**Educational level, n (%)**			
		Lower than a bachelor’s degree	15 (35.7)	
		Bachelor’s degree or higher	27 (64.3)	
	Employed, n (%)			24 (57.1)
	Insured, n (%)			39 (92.9)
	**Income (US $), n (%)**			
		≤50,000	18 (42.9)	
		50,001-99,999	14 (33.3)	
		≥100,000	10 (23.8)	
	**BMI (kg/m^2^), mean (SD)**			29.7 (8.2)
		Normal, n (%)	11 (26.2)	
		Overweight, n (%)	15 (35.7)	
		Obese, n (%)	15 (35.7)	
**Sleep characteristics, n (%)**				
	Short sleep (<7 hours per night)			34 (81.0)
	Poor sleep quality (Pittsburgh Sleep Quality Index>5)			41 (97.6)
	Clinical insomnia (Insomnia Severity Index>14)			31 (73.8)
	Excessive daytime sleepiness (Epworth Sleepiness Scale>10)			20 (47.6)
	History of taking sleep medications			12 (28.6)

### Engagement With the Chatbot

Participants engaged with the chatbot for an average of 7 days over the 2-week intervention (median 7, IQR 5-8, range 4-13 days). On average, they spent 58 minutes interacting with the chatbot (median 49, IQR 27-69, range 9-201 minutes), with each chat session lasting approximately 9 minutes per day (median 7, IQR 4-11, range 1-36 minutes). Correlations between daily chatbot use and each outcome variable are presented in Multimedia Appendix 3. In brief, satisfaction with the chatbot was the only variable that showed a strong correlation with daily use time (*r*=0.39; *P*=.01).

### Usability

**Figure 4:**
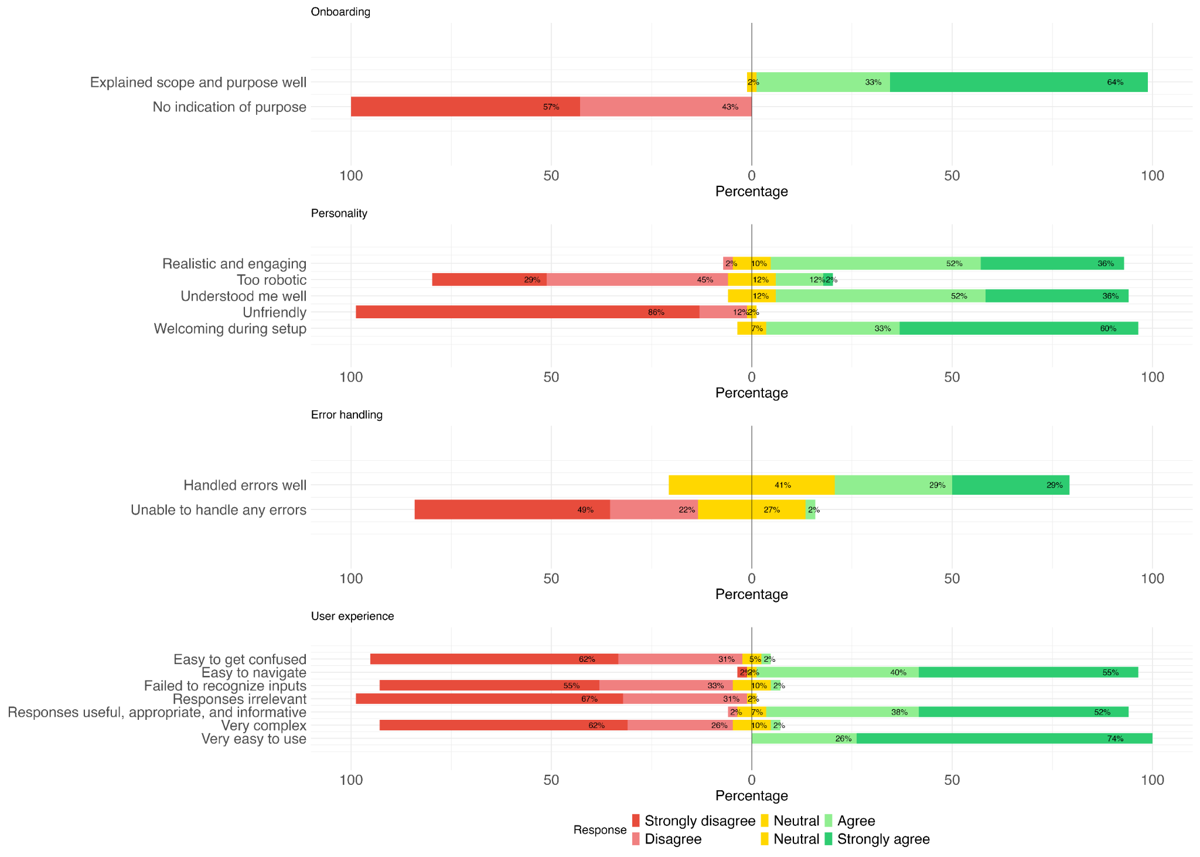
Survey responses to the Chatbot Usability Questionnaire.

The average CUQ score was 85.2 (SD 10.7), indicating a high level of usability compared to the benchmark score of 68. Usability was further assessed across 4 CUQ domains (Figure 4). The onboarding experience received positive feedback, with 98% (41/42) of participants agreeing that the chatbot clearly explained its scope and purpose, and no one felt that the chatbot failed to indicate its purpose. Regarding personality, 88% (37/42) to 98% (41/42) agreed that the chatbot was realistic, welcoming, able to understand them, and friendly, although 14% (6/42) felt that it was somewhat robotic. Participants also reported favorable overall experiences, with 81% (34/42) to 100% (42/42) indicating that the chatbot was easy to navigate and use and that it provided useful and appropriate responses. Only 2% (1/42) found the chatbot complex. In contrast, responses regarding error handling were mixed. There were 57% (24/42) of participants indicating that the chatbot handled mistakes well, 40% (17/42) remained neutral, and 2% (1/42) believed that it was unable to manage errors effectively.

### Acceptability

**Figure 5:**
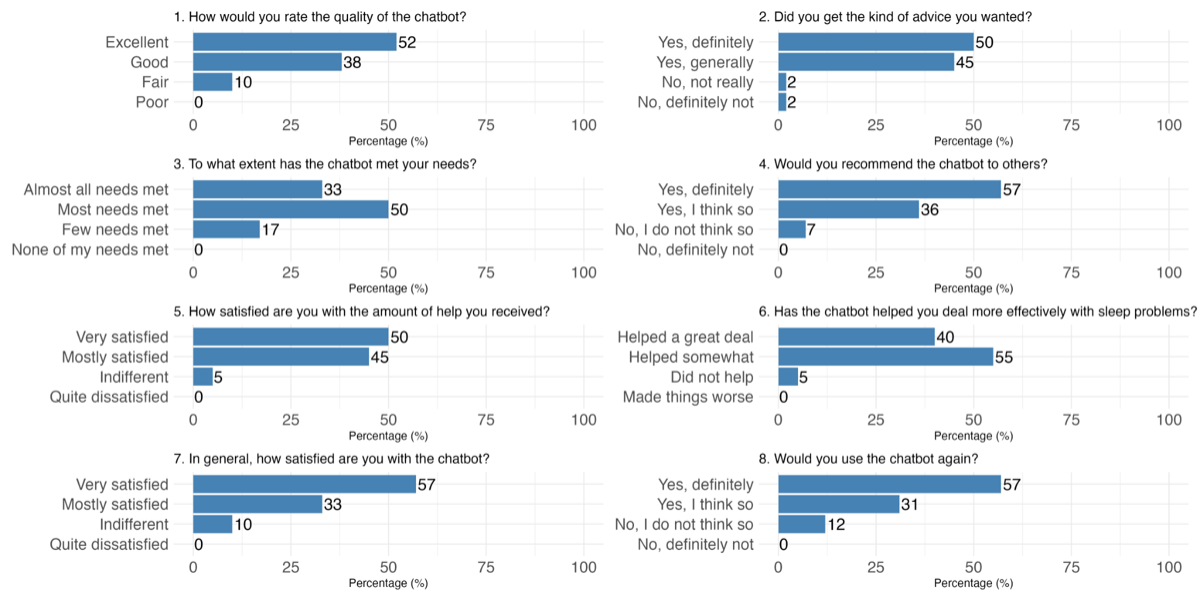
Survey responses to the Client Satisfaction Questionnaire.

The participants found the chatbot highly acceptable, providing an average adapted CSQ score of 27.3 (SD 4.1). Most (38/42, 90%) expressed overall satisfaction with the chatbot. The chatbot’s educational content and advice were well received, with 95% (40/42) agreeing that it helped them manage sleep problems more effectively. Most participants (35/42, 83%) found that their needs were met: 50% (21/42) reported “most needs met,” and 33% (14/42) reported “almost all needs met.” In total, 88% (37/42) indicated that they would use the chatbot again, and 93% (39/42) would recommend it to others (Figure 5).

**Figure 6:**
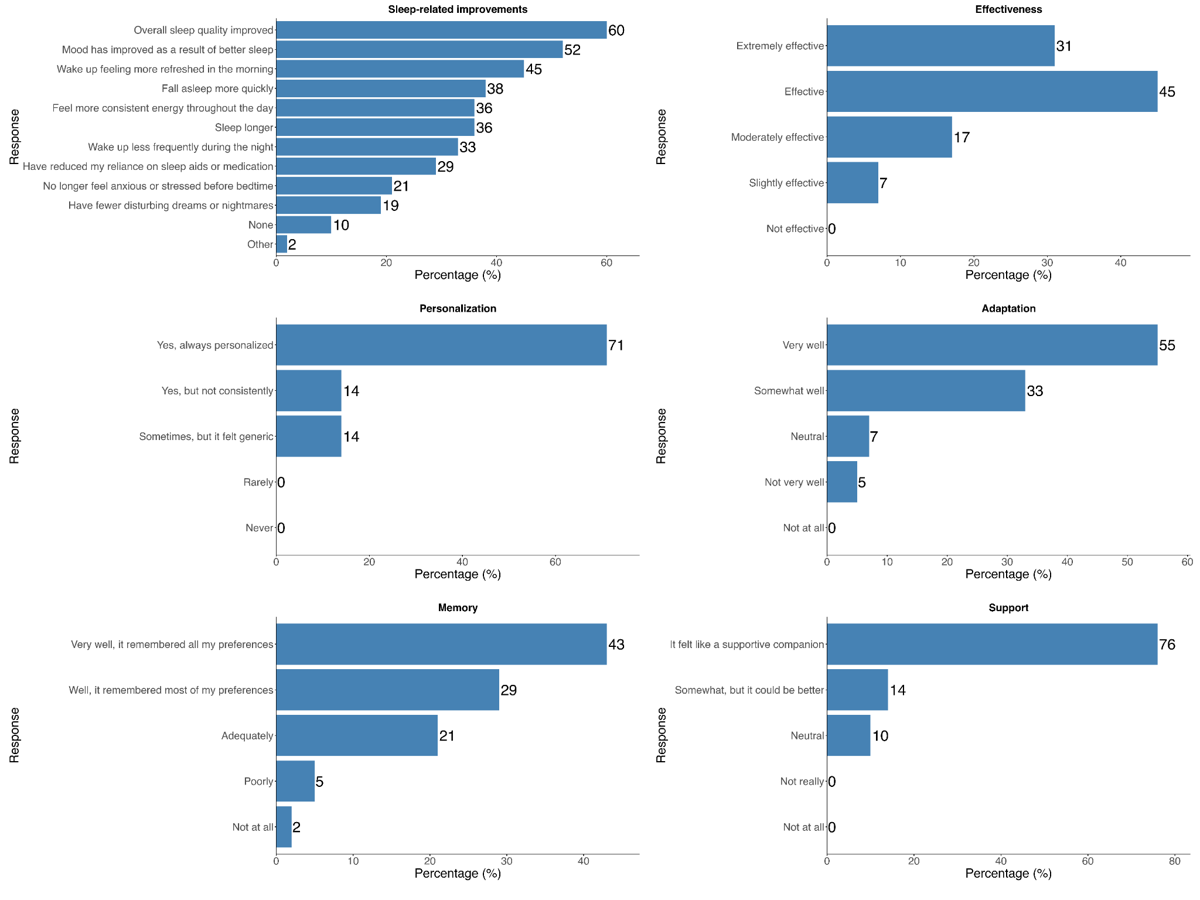
Acceptability of the chatbot.

Participants expressed improvements in several sleep-related areas. The most frequently reported improvement was overall sleep quality (25/42, 60%), followed by mood enhancement (22/42, 52%) and feeling more refreshed in the morning (19/42, 45%). Over one-third reported falling asleep more quickly (16/42, 38%), experiencing more consistent energy throughout the day (15/42, 36%), sleeping longer (15/42, 36%), and waking less frequently during the night (14/42, 33%). All participants considered the chatbot effective, with ratings ranging from “slightly effective” to “extremely effective.” A total of 71% (30/42) reported that the chatbot always personalized its advice, 14% (6/42) felt that personalization was inconsistent, and another 14% (6/42) perceived the responses as generic. Most participants (37/42, 88%) reported that the chatbot adapted its responses to their needs somewhat to very well; however, 5% (2/42) indicated that it did so not very well. A total of 93% (39/42) said that the chatbot was able to remember previous conversations, although 7% (3/42) reported poor or no recall. While most participants viewed the chatbot as a supportive companion, 24% (10/42) felt that its level of support could be better (Figure 6).

### Preliminary Efficacy

**Figure 7:**
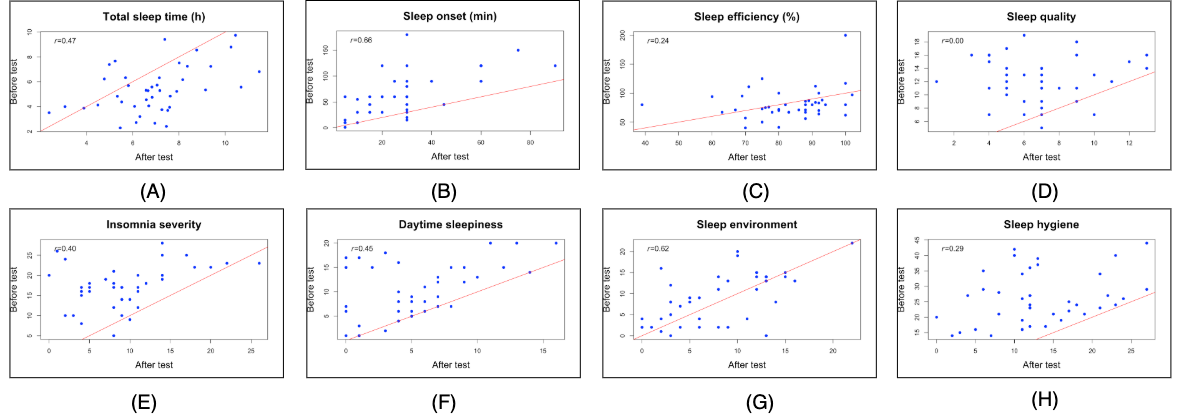
Pre- and postintervention differences in sleep measurements. The red line indicates the diagonal line of equality (y = x). Each blue dot represents an individual participant. Dots below the line indicate higher posttest values than pretest values, whereas dots above the line indicate lower posttest values than pretest values. r indicates the pre-posttest correlation for each sleep measure. Panels show (A) total sleep time, (B) sleep onset, (C) sleep efficiency, (D) sleep quality, (E) insomnia severity, (F) daytime sleepiness, (G) sleep environment, and (H) sleep hygiene.

The sample reported an average total sleep time of 5.3 hours, a sleep onset of 59.5 minutes, and a sleep efficiency of 76.2% at baseline. Following the intervention, changes were observed in all 3 habitual sleep metrics: total sleep time increased by 1.4 hours (*P*<.001), sleep onset decreased by 30.9 minutes (*P*<.001), and sleep efficiency increased by 7.8% (*P*=.007). We also found reductions in the scores of sleep quality (mean difference [MD] 5.4; *P*<.001), insomnia severity (MD -7.9; *P*<.001), daytime sleepiness (MD −4.7; *P*<.001), and sleep hygiene practices (MD −13.2; *P*<.001). There was no statistically significant difference in sleep environment scores (MD −1.1; *P*=.16) before and after the intervention. The proportion of participants who showed improvement in sleep measurements ranged from 60% (25/42; sleep environment) to 100% (42/42; sleep hygiene practices). The pre- and postintervention changes in sleep measurements are shown in Table 2 and Figure 7.

**Table 2:** Differences in sleep measurements before and after the intervention (n=42).

Sleep variables			Pretest time point, mean (SD)		Posttest time point, mean (SD)		Pretest-posttest differences							Participants improved, n (%)
							Mean difference (SD; 95% CI)		*P* value		Cohen *d*			
**Nocturnal sleep metrics**														
	Total sleep time (h)	5.3 (1.5)		6.7 (1.3)		1.4 (1.4; 1.0 to 2.0)		<.001		0.99		30 (71.4)		
	Sleep onset^a^ (min)	59.5 (38.3)		28.7 (20.0)		−30.9 (27.5; −39.6 to −22.2)		<.001		−0.83		34 (82.9)		
	Sleep efficiency^a^ (%)	76.2 (17.0)		84.0 (10.5)		7.8 (16.8; 2.2 to 13.3)		.007		0.54		30 (78.9)		
Sleep quality (0-21)			12.2 (3.4)		6.8 (2.6)		−5.4 (4.3; −6.7 to −4.0)		<.001		−1.77		38 (90.5)	
Insomnia severity (0-28)			17.3 (5.1)		9.4 (5.7)		−7.9 (6.0; −9.8 to −6.0)		<.001		−1.45		39 (92.9)	
Daytime sleepiness (0-24)			10.1 (5.6)		5.4 (4.1)		−4.7 (5.2; −8.5 to −4.0)		<.001		−0.95		30 (71.4)	
Sleep environment (0-39)			9.2 (6.0)		8.1 (5.3)		−1.1 (4.9; −2.6 to 0.4)		.16		−0.19		25 (59.5)	
Sleep hygiene (0-52)			26.2 (8.4)		13.0 (6.6)		−13.2 (9.1; −16.0 to −9.5)		<.001		−1.72		42 (100.0)	

^a^One outlier was removed for sleep onset for analysis (n=41), and 4 outliers were removed for sleep efficiency for analysis (n=38). All variables were assessed for normality using the Shapiro-Wilk test. Because sleep onset, daytime sleepiness, and sleep hygiene violated normality assumptions, we used the rank-based Wilcoxon signed rank test to examine the pretest-posttest differences.

## Discussion

### Principal Findings

To the best of our knowledge, this study was the first to assess a generative AI–based sleep chatbot intervention. Our findings support the feasibility, usability, and acceptability of this prototype AI chatbot. Improvements were found in habitual sleep patterns, subjective sleep quality, severity of insomnia, daytime sleepiness, and sleep hygiene practices after engaging with the chatbot for 2 weeks. Our study highlights the potential of leveraging AI technologies in behavioral interventions to enhance sleep behaviors and outcomes.

Our AI chatbot was feasible in terms of implementation and user engagement. An 82.2% (88/107) sign-up rate among consenting participants suggests that the setup process, including downloading the messaging app and registering for the chatbot, was clear and easy to follow. However, the overall uptake and adherence rates were 60.7% (65/107) and 41.1% (44/107), respectively, which were slightly below expectations. This was because participants had to initiate conversations to start the intervention, an important step that we did not emphasize clearly in this study. Many waited for the chatbot to reach out, leading to missed interactions and reducing the overall adherence. To improve future study retention, we plan to provide clearer instructions and introduce regular check-ins to increase the adherence rate. Encouragingly, participants who completed the 2-week intervention demonstrated high levels of engagement. On average, they interacted with the chatbot for 7 days, which was more frequent than the instructed minimum of once every 3 days. Throughout the study, our participants spent nearly an hour in total using the chatbot, with an average of 9 minutes per day. Although evidence on engagement with sleep chatbots is scarce, we found similar engagement rates with chatbots developed for other health behaviors. For example, one study reported an average chatbot use of 5.1 minutes per day for promoting physical activity [28]. Another study found that adolescents spent approximately 45 minutes using a health education chatbot, with sessions averaging 4 minutes [29]. Nevertheless, caution should be taken when comparing engagement rates across chatbot interventions due to variations in study focus, design, duration, and mode of delivery.

Participants reported positive experiences with the chatbot. They found the chatbot easy to use, appreciated its realistic and welcoming tone, and considered it helpful. Participants also expressed satisfaction with the chatbot’s personalized and adaptive content. In general, the level of usability and acceptability of our chatbot was higher than that reported in previous research. A systematic review by Aggarwal et al [13] found that users rated chatbot interventions as having low to moderate ease of use. Additionally, fewer than 50% of users reported satisfaction with the chatbot [13]. The favorable outcomes in our study were primarily driven by the generative AI technology. LLMs are inherently adaptive, enhancing participant engagement by simulating realistic human conversations and allowing for personalized learning through reinforcement learning. As participants interact, the LLMs progressively refine responses to enhance their performance as individual data accumulate. This interactive process enables the models to update sleep recommendations in real time, which is a feature unachievable with traditional sleep interventions. Despite overall high satisfaction, several areas for improvement were identified. Some participants perceived the chatbot’s responses as generic or robotic, highlighting the need for further training of LLMs to promote more natural and coherent interactions. In addition, enhancing the chatbot’s memory capabilities is critical to maintain conversational continuity and deliver more personalized, contextually relevant responses.

Our findings provide preliminary evidence of sleep improvement after chatbot use. The study results echoed prior evidence on the use of conversational agents for monitoring and promoting sleep. Werner-Seidler et al [30] evaluated a 6-week app-based program that delivered CBT-I through text–based interactions with a virtual sleep therapist. Compared to the control group that received weekly sleep tips via texting, the intervention group experienced significantly greater reductions in insomnia severity and better sleep quality after the intervention and 2-month follow-up [30]. Similarly, another study developed 2 apps and randomly assigned adults with insomnia to either a control group (sleep diary only) or an intervention group (personalized recommendations from a virtual companion) [31]. After 17 days, the intervention group exhibited reduced insomnia severity, gained 48 additional minutes of sleep per night, increased sleep efficiency, and decreased wake after sleep onset (*P*<.05) compared to the control group [31]. Although our chatbot achieved comparable improvements within a shorter time frame, the study findings should be interpreted with caution because all sleep outcomes were assessed via questionnaires. Discrepancies between subjective and objective sleep measurements are well documented [32,33]. One study found that self-reporting overestimated sleep duration by 64 minutes compared with actigraphy- and polysomnography-measured sleep [32]. We acknowledge that the observed sleep improvements could be subject to reporting bias. In our next trial, we will include wrist actigraphy to obtain a more accurate, comprehensive evaluation of sleep outcomes.

Several additional features of the AI chatbot support its potential for broader implementation and scalability. First, it is cost-effective. Traditional individual sleep therapy sessions such as CBT-I cost between US $750 and US $2500 [34]. As some insurance plans do not cover CBT-I treatments, the out-of-pocket expenses often pose financial challenges for many individuals struggling with sleep problems. In contrast, our AI chatbot is fully automated, substantially reducing the need for human resources. Unlike previous digital sleep interventions that required considerable local storage and computing power for machine learning models and data processing, our chatbot operates via LLMs hosted on cloud servers, eliminating the need for high-performance hardware. Additionally, the use of LLMs allows for seamless integration into a variety of software systems, which minimizes costs associated with app development and maintenance. Furthermore, the AI chatbot is highly accessible. Access to sleep therapy has been a long-standing issue because it is often administered by clinical specialists in person or in group settings. This issue is more notable in regions (eg, rural areas) with a shortage of trained health care providers [35]. Deploying the chatbot through a widely used commercial texting app eliminates the need for participants to learn new technology or undergo technical use training. The texting app’s simple, intuitive interface enables individuals with basic smartphone skills to seek sleep support anytime and anywhere. Using a private, text–based platform can also help reduce the stigma often associated with in-person therapy, an important factor to boost engagement.

### Limitations

Despite the aforementioned advantages, our study has several limitations. First, as the quasi-experimental design cannot infer causality, a randomized controlled trial needs to be conducted to confirm the study findings. Second, we did not examine the long-term effects of the intervention. Future studies should include follow-up assessments to evaluate the sustainability of sleep improvements. Third, although participants were recruited nationwide, the study is underpowered due to its small sample size. Additionally, convenience sampling contributed to sociodemographic imbalances (eg, age, race, and educational attainment). These factors introduced selection bias and may limit the generalizability of the findings. In our next trial, we will use stratified sampling to achieve a more balanced and representative cohort. Fourth, the intervention delivery mode may exclude certain populations, such as individuals who have insufficient smartphone technological skills, who do not own a smartphone, or who are unwilling to engage with a chatbot through texting. Finally, sleep was assessed only using self-report. Prospective work should integrate objective sleep assessments to provide a more comprehensive examination of the intervention effects.

### Conclusions

Our study demonstrates that an AI-powered, text–based chatbot is a feasible, usable, and highly acceptable intervention for adults with poor sleep. The findings provide preliminary evidence of the association between chatbot use and improved sleep outcomes. Building on the study results, future research will refine the chatbot’s design and functionality based on the limitations identified in this study. Rigorous evaluation through randomized controlled trials is necessary to validate the study findings and establish causality. If confirmed, the chatbot’s affordability, engaging interface, and personalized features position it as a potential tool for large-scale implementation.

## Appendix

Multimedia Appendix 1 Model configuration and safety settings of generative artificial intelligence.

Multimedia Appendix 2 Comparison of sociodemographic and clinical characteristics between completers and noncompleters.

Multimedia Appendix 3 Correlations between chatbot daily use time and study variables.

## Abbreviations

AI: artificial intelligence
CBT-I: cognitive behavioral therapy for insomnia
CSQ: Client Satisfaction Questionnaire
CUQ: Chatbot Usability Questionnaire
LLM: large language model
MD: mean difference

## Data Availability

The datasets generated or analyzed during this study are not publicly available due to the protection of participants’ privacy and confidentiality but are available from the corresponding author upon reasonable request.
