# Supplementary material for: Assessing the Feasibility, Usability, Acceptability, and Efficacy of an AI Chatbot for Sleep Promotion: Quasi-Experimental Study
Source: JMIR Form Res. 2026 Feb 3;10:e84023. doi: 10.2196/84023 (PMC12914230; doi:10.2196/84023)
Supplement: Multimedia Appendix 1 [file formative_v10i1e84023_app1.pdf]

Multimedia Appendix 1. Model configuration and safety settings of generative artificial intelligence.

```
generation_config = {
   "max_output_tokens": 8192,
   "temperature": 1,
   "top_p": 0.95,
}
safety_settings = [
   SafetySetting(
      category=SafetySetting.HarmCategory.HARM_CATEGORY_HATE_SPEECH,
      threshold=SafetySetting.HarmBlockThreshold.BLOCK_MEDIUM_AND_ABOVE
   ),
   SafetySetting(

category=SafetySetting.HarmCategory.HARM_CATEGORY_DANGEROUS_CONTENT,
      threshold=SafetySetting.HarmBlockThreshold.BLOCK_MEDIUM_AND_ABOVE
   ),
   SafetySetting(
      category=SafetySetting.HarmCategory.HARM_CATEGORY_SEXUALLY_EXPLICIT,
      threshold=SafetySetting.HarmBlockThreshold.BLOCK_MEDIUM_AND_ABOVE
   ),
   SafetySetting(
      category=SafetySetting.HarmCategory.HARM_CATEGORY_HARASSMENT,
      threshold=SafetySetting.HarmBlockThreshold.BLOCK_MEDIUM_AND_ABOVE
   ),
]
```
