# Supplementary material for: Assessing the Feasibility, Usability, Acceptability, and Efficacy of an AI Chatbot for Sleep Promotion: Quasi-Experimental Study
Source: JMIR Form Res. 2026 Feb 3;10:e84023. doi: 10.2196/84023 (PMC12914230; doi:10.2196/84023)
Supplement: Multimedia Appendix 2 [file formative_v10i1e84023_app2.pdf]

Multimedia Appendix 2. Comparison of sociodemographic and clinical characteristics between completers and noncompleters.

**Variables**	Completers (n = 42)	Non-Completers (n = 46)	*p*
Age in years	36 ± 11	37 ± 12	0.686
Sex			
Male	12 (50%)	12 (50%)	0.790
Female	30 (47%)	34 (53%)	
Race			
White	26 (41%)	37 (59%)	0.091
Non-White	16 (64%)	9 (36%)	
Education			
Below bachelor's degree	15 (38%)	25 (62%)	0.124
Bachelor's degree or above	27 (56%)	21 (44%)	
Employed status	24 (44%)	30 (56%)	0.577
Insured status	39 (50%)	39 (50%)	0.392
Income			
≤ $50,000	18 (49%)	19 (51%)	0.888
$50,001 - $99,999	14 (50%)	14 (50%)	
≥ $100,000	10 (43%)	13 (57%)	
Short sleep (< 7 h/night)	34 (51%)	33 (49%)	0.446
Poor sleep quality (Pittsburgh Sleep Quality Index > 5)	41 (49%)	43 (51%)	0.991
Clinical insomnia (Insomnia Severity Index >14)	31 (53%)	28 (47%)	0.288
Daytime sleepiness (Epworth Sleepiness Scale > 10)	20 (56%)	16 (44%)	0.434
History of taking sleep medications	12 (38%)	20 (62%)	0.219
Note: Data are presented as either mean ± standard deviation or count (percentage)			
