# Supplementary material for: Assessing the Feasibility, Usability, Acceptability, and Efficacy of an AI Chatbot for Sleep Promotion: Quasi-Experimental Study
Source: JMIR Form Res. 2026 Feb 3;10:e84023. doi: 10.2196/84023 (PMC12914230; doi:10.2196/84023)
Supplement: Multimedia Appendix 3 [file formative_v10i1e84023_app3.pdf]

Multimedia Appendix 3. Correlations between chatbot daily use time and study variables.

**Variables**	**Daily Usage Time**	
	**r**	***p***
**Chatbot usability**	0.27	0.086
**Satisfaction with chatbot**	0.39	0.011
**Total sleep time, h**	0.07	0.675
**Sleep onset, min**	0.08	0.620
**Sleep efficiency, %**	0.01	0.961
**Sleep quality**	-0.25	0.105
**Insomnia severity**	-0.25	0.103
**Daytime sleepiness**	-0.24	0.128
**Sleep environment**	-0.12	0.465
**Sleep hygiene**	-0.11	0.504
